# ‘Obesogenic’ School Food Environments? An Urban Case Study in The Netherlands

**DOI:** 10.3390/ijerph15040619

**Published:** 2018-03-28

**Authors:** Joris Timmermans, Coosje Dijkstra, Carlijn Kamphuis, Marlijn Huitink, Egbert van der Zee, Maartje Poelman

**Affiliations:** 1 Department of Human Geography and Spatial Planning, Faculty of Geosciences, Utrecht University, Princetonlaan 8a, 3584 CB Utrecht, The Netherlands; j.m.timmermans@students.uu.nl (J.T.); E.L.vanderZee@uu.nl (E.v.d.Z.); 2 Department of Health Sciences, Faculty of Earth and Life Sciences, Amsterdam Public Health Research Institute, VU-University, De Boelelaan 1085, 1081 HV Amsterdam, The Netherlands; coosje.dijkstra@vu.nl (C.D.); m.huitink@vu.nl (M.H.); 3 Department of Interdisciplinary Social Science, Faculty of Social and Behavioural Sciences, Utrecht University, Heidelberglaan 1, P.O. Box 80140, 3508 TC Utrecht, The Netherlands; C.B.M.Kamphuis@uu.nl

**Keywords:** food environment, obesity, urban areas, retail outlets, food advertisements, secondary school, eating behavior, nutrition, adolescents

## Abstract

(1) Background: This study aimed to explore and define socio-economic (SES) differences in urban school food environments in The Netherlands. (2) Methods: Retail food outlets, ready-to-eat products, in-store food promotions and food advertisements in public space were determined within 400 m walking distance of all secondary schools in the 4th largest city of The Netherlands. Fisher’s exact tests were conducted. (3) Results: In total, 115 retail outlets sold ready-to-eat food and drink products during school hours. Fast food outlets were more often in the vicinity of schools in lower SES (28.6%) than in higher SES areas (11.5%). In general, unhealthy options (e.g., fried snacks, sugar-sweetened beverages (SSB)) were more often for sale, in-store promoted or advertised in comparison with healthy options (e.g., fruit, vegetables, bottled water). Sport/energy drinks were more often for sale, and fried snacks/fries, hamburgers/kebab and SSB were more often promoted or advertised in lower SES areas than in higher SES-areas. (4) Conclusion: In general, unhealthy food options were more often presented than the healthy options, but only a few SES differences were observed. The results, however, imply that efforts in all school areas are needed to make the healthy option the default option during school time.

## 1. Introduction

Worldwide, overweight and obesity among adolescents have increased tremendously over the past decades and are a major health problem [1]. These figures have also increased in The Netherlands [2]. Currently, 15.6% of Dutch adolescents are either overweight or obese [3]. Moreover, there are substantial socioeconomic inequalities in the youth overweight and obesity rates, especially in urban environments [4,5]. Prevalence of obesity within urban areas shows spatial patterns that seem to correlate with the socio-economic status of neighborhoods [6,7]. In Utrecht, the 4th largest city in The Netherlands, the most deprived neighborhood has a childhood obesity rate of 35%, while this is 2% in the least deprived neighborhood [8]. Overweight and obesity arise from an energy imbalance, where energy intake exceeds energy expenditure. Although a complex web of factors contribute to adolescents’ energy intake, the food environment is considered a prominent driver of energy-dense food consumption [9,10].

The food environment can be characterized as the availability, accessibility and promotion of food and drink products in the environment. As adolescents spend a large amount of their time in and around schools, the school food environment is an important setting for adolescents’ food intake and the development of their eating habits [10]. Recently, secondary schools have been pressured to offer and promote healthy foods in their school canteens [11]. However, in the case of The Netherlands, high-school students are not obliged to stay within school boundaries during breaks for the consumption of snacks or lunches. Recently, it has been found that 31% of Dutch adolescents living in urban areas never purchase foods in the school canteen during school time. Moreover, the vast majority (71%) of the adolescents indicate that they purchased foods and drinks from outlets present in the direct surroundings of their school [12]. 

Prior studies confirm that the school food environment is associated with adolescents’ food intake [13,14] and weight status [15]. For example, the number of fast food outlets around schools was positively associated with the body mass index of adolescents [16]. The food environment is often called ‘obesogenic’ because it contributes to behavior associated with obesity (e.g., excessive energy intake) [17]. Furthermore, socioeconomic differences in food outlet availability around schools have been suggested in prior studies [18,19]. Schools located in deprived urban neighborhoods generally have greater access to unhealthy retail outlets [9,19,20,21,22]. For example, schools located in the most deprived urban neighborhoods in New-Zealand had 13.9% more unhealthy retail outlets available than schools in the least deprived areas [9]. Furthermore, previous studies have indicated that unhealthy food advertisements in public space are present within short walking distance of public schools in both low-, middle- and high-income countries [23,24,25,26,27]. In addition to food advertisements in public spaces, marketing and sales-promotions continue within stores and may affect purchase behavior [28]. Although prior studies already provide important knowledge on international school food environments in urban areas, insights in food environmental characteristics in The Netherlands are currently lacking. Moreover, most studies have been limited to either exploring the type of food outlet, or studying the presence of advertisements and the availability of promotions. Since the school food environment consists of all these factors together, it is important to study them in combination to provide a more comprehensive view. 

This study adds to the literature by combining the examination of (1) retail-outlets, (2) ready-to-eat food products, (3) in-store food promotions, and (4) food advertisements in public space in the direct surroundings of schools in an urban setting in The Netherlands. The secondary aim of this study is to determine to determine differences in these food environmental attributes with respect to the socioeconomic status of the neighborhood environment in which the school is located. 

## 2. Materials and Methods 

### 2.1. Study Design and Setting 

**Figure 1:**
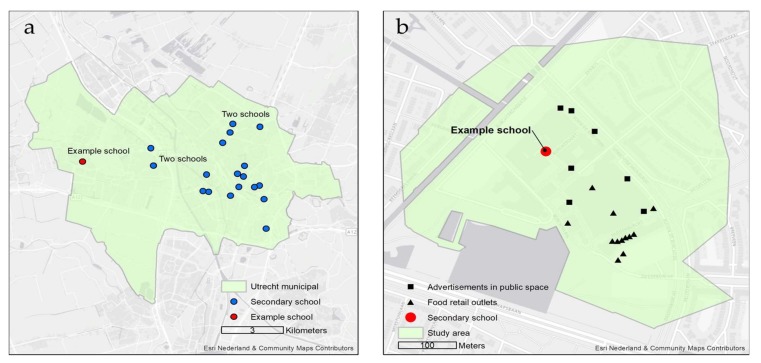
(**a**) All secondary school locations in the Utrecht municipality (*N* = 21); (**b**) Example of a school food environment around one secondary school including food retail outlets and public food advertisements.

A cross-sectional observational study was conducted in the municipality of Utrecht, The Netherlands, in November and December 2016. Utrecht has a population of approximately 330,000 residents and accommodated 21 secondary schools in 2016, hosting a total of 15,426 students. Figure 1 presents an overview of the school locations and one example of a school food environment surrounding a secondary school [29]. In this study, the school food environment was defined as the 400 m road network buffer surrounding the secondary school. Based on prior research, this distance was chosen because it provides a general measure of the area students are able to access on foot during a short (lunch) break of 15 min [30]. 

### 2.2. Study Procedure

Retail food outlets present within the 400-m buffer of each secondary school were determined by means of the Locatus database [31], maintaining independently sourced retail information via annual on-site surveys. Data on the location and type of retail-food outlets (Table 1) within the municipality of Utrecht throughout 2015 were extracted. Subsequently, additional food environmental characteristics were collected by visiting all secondary school 400-buffer areas using an observational checklist especially developed for this study purpose. This checklist was based on existing surveys to define food assortments and advertisements [10,32,33], but was adapted to the Dutch situation and study purpose. By means of the checklist, we determined the availability (yes/no) and type of ready-to-eat food products, in-store food promotions and public food advertisements, and differentiated for healthy and unhealthy food and drink products. The checklist was developed and pilot-tested by (J.T, C.D. and M.P.) in a non-school environment. Observations with this checklist were conducted by one of the researchers (J.T.) who visited all school food environments of the 21 included schools. Appendix A includes the checklist, and in Section 2.3, checklist measurements are explained in more detail. 

### 2.3. Data and Measures

#### 2.3.1. Retail Food Outlets (Type and Healthiness) 

In this study, retail food outlets were defined as all places selling ready-to-eat food during regular school hours (10 a.m.–3 p.m.). Not only typical food outlets (e.g., fast food outlets, supermarkets) were taken into account, but also stores whose core business was not selling food (e.g., chemists), but that sell ready-to-eat food. During the observations of the school food environments, outlets were verified by name, type, and location. In this way, possible discrepancies between the Locatus database and the observed food retail outlets were checked. In total, five outlets were altered due to incorrect data in the Locatus database.

In addition to the type of food outlet that was taken into account, a corresponding ‘healthiness score’, ranging from −5 (very unhealthy) to +5 (very healthy), was determined for each food outlet. For example, a fast-food outlet was scored −4.9, whereas a green-grocer had a score of 4.8. Table 1 presents an overview of the healthiness score of each food retail outlet. These healthiness scores were included to avoid a dichotomous categorization of retail outlets into either ‘healthy’ or ‘unhealthy’ and to give a more nuanced indication of the healthiness of school food environments, based on the overall number of retailers selling food. Based on the methodology of and in line with international research [34,35], the healthiness score was derived from an online Delphi study including 20 academics and nutrition experts from The Netherlands working in the field of public health nutrition, epidemiology, health science or eating psychology [36]. In several (anonymous) rounds, scores and rationales for each outlet were provided by each expert. Subsequently, the range of scores and rationales were shared among all experts and discussed. This final step was repeated until consensus was reached.

#### 2.3.2. Ready-to-Eat Food Assortment and In-Store Promotions 

For each retail food outlet, it was determined what types of ready-to-eat food products were for sale. Moreover, it was examined whether these products were promoted or for sale within the shops. The ready-to-eat food assortment included food and drink categories often bought by adolescents during school time [12,37]. The assortment included twelve product categories that were classified as either healthy or unhealthy, according to the guidelines of The Netherlands Nutrition Centre [38]. These guidelines indicate that drinks containing more than 5 g of sugar per 100 milliliters, or food consisting of either 20 percent sugar or saturated fats contribute to an unhealthy diet. Availability and in-store promotions of seven unhealthy and five healthy food categories were determined. Unhealthy food and drink product categories included savory pastries (e.g., sausage roll, croissant), fried snacks (e.g., chips, croquettes), hamburger/kebab, sweets and savory snacks, pizza, sugar sweetened beverages (SSB), sport/energy drinks and fruit juices were classified as unhealthy. Healthy food and drink product categories were fruit (single pieces or fruit salad), vegetable snacks (e.g., mini-cucumbers or cherry tomatoes), bottled water, lite (soft) drinks (e.g., diet coke) and non-sugared dairy (e.g., milk).

#### 2.3.3. Public Food Advertisements

For all school food environments, food advertisements in public spaces were observed and counted. Food advertisements were defined as adverts promoting retail food outlets, food brands or specific food products or meals. All advertisements on stationary objects (i.e., wall posters, banners, bus-stop advertisements, flags, free-standing signs) in public space were included. Shop windows promoting foods or brands were excluded. Additionally, stationary delivery vehicles promoting retail outlets, food brands or specific food products or meals were included (e.g., parked delivery vehicles). For every advertisement in the public space, a picture with a global positioning system (GPS) tag was taken, and food advertisements were categorized. First, food advertisements were categorized into ready-to-eat food products (e.g., French fries, pizza) vs. food products that need to be prepared prior to consumption (e.g., potatoes, minced meat). Subsequently, food advertisements were categorized as being healthy or unhealthy using the similar categories as for the ready-to-eat food assortment. 

#### 2.3.4. Neighborhood Socioeconomic Status and Commercialization 

To identify socioeconomic differences in the school food environment, we took neighborhood socioeconomic status (SES) into account by using socioeconomic scores derived from The Netherlands Institute for Social Research (NISR, https://www.scp.nl/), providing a general measure of neighborhood SES. The NISR created the score per four-digit zip code using a principal component analysis, based on the education, income and labor market positions of its inhabitants, with scores ranging from −2.02 to 2.11. The SES score for the neighborhood of each school was calculated. Subsequently, the median SES score of The Netherlands (0.78) was used as a cutoff point to categorize school neighborhoods into either lower or higher SES areas. 

To account for commercialization in SES difference in school food environments, we also studied the association between commercialization and the main outcome measures. Commercialization was determined by retail location density per four-digit zip code, and was derived from the Dutch Neighborhood Statistics 2016 and used to determine commercialization of the school food environment [39]. Values ranged from 36 to 1911 retail locations per square kilometer, categories (lower vs. higher commercialized areas) were split at the urban median value of 96 retail outlets per square kilometer.

### 2.4. Spatial and Statistical Data Analysis

Location data was processed using ESRI’s ArcGIS for Desktop 10.3 (ESRI, Redlands, CA USA), and statistical analyses were conducted using IBM SPSS Statistics 24 (IBM, North Castle, NY, USA). The number of retail outlets, ready-to-eat food assortment and public food advertisements within this buffer were determined. To determine the average number of retail outlets, first all schools were included (*N* = 21), and thereafter only schools with retail food outlets in its 400 buffer (*N* = 14) were included. For each school food environment, the number of retail food outlets, ready-to-eat food products and food advertisements were determined by means of descriptive statistics. Moreover, an overall healthiness sum score including all the retailers that offered ready-to-eat food products during school time was calculated for each school. An independent samples t-test was conducted to examine SES differences in the overall healthiness score. To determine differences in the percentages of retail food outlets, ready-to-eat food assortment and food advertisements between low and high neighborhood SES, Fisher’s exact tests were conducted. Similar analyses were conducted to determine the potential interference of commercialization in the association between SES and the school food environment outcome measures. 

## 3. Results

### 3.1. Retail Outlets Selling Food

During data collection, 285 retail outlets were observed. In total, 117 (41.7%) retail outlets were closed during school lunch hours, and 53 (18.0%) outlets did not sell any ready-to-eat food products. Therefore, in total, 115 (40.3%) retail food outlets were included for analyses in this study. For seven secondary schools (33.3%), no retail food outlets were present within 400 m. 

As can be seen in Table 1, the most common retail outlets in the school food environment were lunchrooms (17.4%) and fast food retail outlets (15.7%). As a proportion of the total number of food stores available, fast food retail outlets were more often present in lower SES than in higher SES school environments (28.6% vs. 11.5%, *p* = 0.04, Fisher’s exact test), whereas the opposite was found for lunchrooms (21.8% (higher SES) vs. 3.6% (lower SES), *p* = 0.04, Fisher’s exact test). These differences were not affected by school neighborhood commercialization rate (Appendix B). The average retail healthiness score for the 14 secondary school food environments who offered food was −16.9 (SD = 17.5), and varied between −62 and −1. The retail healthiness scores did not statistically significant differ between low (m = −15.3 SD = 12.3) and high (m = −17.9 SD = 21.8) SES school environments (t = −0.469, *p* = 0.64).

### 3.2. Ready-to-Eat Food Assortment 

As can be seen in Table 2, the most frequently available products for sale during school time in the school environment were drinks. Although lite soft drinks (77.4%) and bottled water (67.0%) were for sale in the majority of retail outlets, SSBs (84.3%) were predominantly offered. Energy/sport drinks were for sale in 43.5% of the retail outlets. Fruit and vegetable snacks were for sale during school time, but in the minority of retail outlets (23.5% and 12.2%, respectively). Mainly unhealthy food products were promoted during the time of data collection. For example, water was promoted in 3.5% of the outlets, whereas this was 27.8% for SSBs. 

Sport and energy drinks were more often for sale in lower than in higher socioeconomic school environments (66.7% vs. 35.6%, *p* = 0.004, Fisher’s exact test). Fried snacks and fries were also more often in-store promoted in low than in high SES school environments (25.0% vs. 9.2%, *p* = 0.05, Fisher’s exact test). In higher socioeconomic school environments, savory pastries were more often for sale than lower socioeconomic school environments (59.8% vs. 37.0%, *p* = 0.03, Fisher’s exact test, Table 3). No differences in these sales figures and in-store promotion rates were found for commercialization rate (Appendix B).

### 3.3. Public Food Advertisements 

A total of 350 advertisements in public space were observed in the school environments, of which 143 (40.9%) advertisements contained food products. The average number of public food advertisements was 6.81 (SD = 8.60) per school. Yet, public food advertisements were present in only 14 (66.6%) schools, although these were not the same 14 schools that contained retail food outlets. When including only these 14 schools, the average number public food advertisements was 11.14 (SD = 17.65). As indicated by Table 2, a substantial percentage of these food advertisements (26.6%) targeted food products that were not characterized as ready-to-eat (e.g., minced meat).

Of public food advertisements, unhealthy food advertisements (58.0%) were more common than healthy advertisements (13.7%, Table 2). The most advertised food products were SSBs (13.7%), the least advertised food products were sport and energy drinks (0.6%), and water (0.6%). 

Sugar-sweetened beverages (22.5% vs. 6.0%, *p* = 0.02, Fisher’s exact test) and hamburgers/kebab (22.5% vs. 7.7%, *p* = 0.04, Fisher’s exact test) were more often advertised in lower than in higher socioeconomic school environments. These differences were not affected by degree of school neighborhood commercialization (Appendix C). 

## 4. Discussion

The results of this study show that unhealthy food and drink products were predominantly for sale and promoted in secondary school areas in the municipality of Utrecht. Fruit was for sale in less than a quarter of the outlets around the schools (23.5%), and this was even less for vegetable snacks (12.2%). SSBs (84.3%) were more often available than lite drinks (77.4%) or bottled water (76.0%). There was no difference between lower and higher SES areas in the overall healthiness score of the school food environment. Yet, fast food outlets were more often in the vicinity of secondary schools in lower SES neighborhoods (28.6%) than in higher SES neighborhoods (11.5%). Sport/energy drinks were more often for sale, fried snacks/fries more often in-store promoted and SSB and hamburgers/kebab more often advertised in lower than in higher SES school environments. Overall, this study suggests that unhealthy food is the default in urban school food environments, and implies that this is somewhat more the case in lower SES neighborhoods. 

These results are in line with previous international studies comparing low and high socioeconomic school environments [9,19,20,21,22]. For example, in Victoria, Australia, at least one fast food outlet was located within 80% of the most deprived school environments, vs. 29% in the least deprived school environments [19]. A nationwide study in the US reported that schools located in low-income neighborhoods had 32% more fast food retail outlets than those in high-income neighborhoods [22]. Yet, it is important to highlight that these studies included rather large study areas in comparison to the present one-city-focused study. In our study, the overall healthiness score did not differ between low and high socioeconomic school environments. Furthermore, in a prior study in The Netherlands, no SES difference in the accessibility of healthy and fresh food via supermarkets in urban areas was found [40]. 

A recent study [41] in the city of Santos in Brazil explored the home food environment of children (<10 y). In this study, in-store foods were categorized by the degree of industrial processing. Results indicate that 60.6% of the retail food outlets had the highest proportion of ready-to-eat food products (processed or ultra-processed) for sale, not facilitating healthy food consumption patterns. A New Zealand study also found that the majority of advertisements in urban school environments were for food products that should be provided or sold in limited quantities during school time (e.g., SSBs, fried snacks) [24]. Although not specifically targeted at school environments, studies have indicated that the majority of food marketing is for food products unfavorable for maintenance of a healthy diet or the promotion of a healthy weight. For example, 18% of promoted products in Australian supermarkets met the accepted nutrition standards for foods sold to youth [42]. A more general study in The Netherlands also revealed similar trends: 70% of food promotions were categorized as unhealthy [43]. All in all, these results indicate that, in line with other countries, urban Dutch food environments surrounding the school are also not favorable to stimulating healthy choices. 

Strengths of the study include the use of both a GIS-database analyses and on-site observations. Moreover, we extended the measurement of the school food environments by including the observation of ready-to-eat food products, in-store food promotions and food advertisements in public space. This provides an in-depth scrutiny of school food environments, combining multiple indicators. Weaknesses of the study include the inclusion of a rather small urban environment of The Netherlands as only the municipality of Utrecht was included. Yet, data-collection via observations of the food that was offered, in-store food promotions and advertisements in all the school environments was a rather intensive and time-consuming procedure. Another limitation is the cross-sectional character of the study. In-store promotions and food advertisements in public space, in particular, are subject to temporal changes that were not captured by means of this study. Moreover, the healthiness score used is based on expert opinions, remaining a subjective measure of indexing the healthiness of the food environment. 

Future research should include a larger area, and also compare the school food environment of more rural areas with those of urban areas. Additionally, including a more sensitive SES measure—including more area-level SES categories—may provide more detailed insight into food environmental SES differences. Moreover, monitoring school food environments by means of longitudinal measurements is desirable. In doing so, public health efforts to improve the school food environment of the youth can also be monitored. This allows us to understand whether such efforts actually result in healthier food environments. Additionally, next to retail food outlets, ready-to-eat products, in-store promotions (e.g., discounts) and public food advertisements, additional food environmental characteristics should be included. For example, food prices could be measured, especially since the price of healthy products has increased more in comparison to the price of unhealthy products [44]. Another recommendation for future research is to better understand the environment-diet relationship. It is understood that unhealthy food environments are unbeneficial for healthy food decisions, but the interaction between the individual and the food environment, and the underlying mechanisms for this, is not well understood.

These results show that the urban school food environment is predominantly unhealthy and may compete with the efforts of public health professionals to create a healthy school environment, such as the Healthy School Canteen Programme in The Netherlands. These efforts are mitigated if students purchase snacks or lunches elsewhere. Therefore, efforts in the wider environment may be desirable. In Amsterdam, The Netherlands, the network ‘Entrepreneurs for a Healthy Amsterdam’ was established, in which urban retail owners unite to develop and sell healthy foods to the Amsterdam youth. Additionally, more firm actions have been proposed. For example, the mayor of London, UK, announced a fast-food outlet ban in school areas to attenuate the influence of these corporations on the eating behaviors of the London youth. 

## 5. Conclusions

This research is the first to combine the examination of (1) retail food outlets, (2) ready-to-eat food products, (3) in-store food promotions, and (4) public food advertisements in school food environments in an urban setting. For all of these indicators, the unhealthy options were more often presented than the healthy options, but only a few SES differences were observed. Future research is needed to better understand students’ interaction with the food environment surrounding schools. However, the figures presented imply that efforts to support healthy school food environments are needed to make the healthy option the default option during school time.

## Figures and Tables

**Table 1:** Retail outlets in 400-m buffers surrounding secondary schools in the municipality of Utrecht, The Netherlands.

Type (Healthiness Score)	Total		Neighborhood Socioeconomic Status			
			Low		High	
	*N*	%	*N*	%	*N*	%
Fast-food (−4.9)	18	15.7	**8**	**28.6**	**10**	**11.5**
Grillroom (−4.8)	4	3.5	2	7.1	2	2.3
Liquor store (−4.6)	7	5.2	2	7.1	4	4.5
Petrol station (−4.5)	1	0.9	0	0.0	1	1.1
Chocolate store (−4.3)	3	2.6	0	0.0	3	3.4
Drugstore (−4.0)	9	7.8	4	14.3	5	5.7
Ice-cream store (−3.9)	2	1.7	1	3.6	1	1.1
Take away (−3.7)	8	7.0	1	3.6	7	8.0
Café-restaurant (−1.9)	6	5.2	0	0	6	6.8
Lunchroom (−1.5)	20	17.4	**1**	**3.6**	**19**	**21.8**
Butcher (−0.4)	2	1.7	0	0.0	2	2.3
Delicacies (0.2)	3	2.6	0	0.0	3	3.4
Mini super (0.3)	4	3.5	1	3.6	3	3.4
Reform shop (1)	2	1.7	0	0.0	2	2.3
Bakery (1.2)	8	7.0	2	7.1	6	6.9
Coffee/tea store (1.3)	4	3.5	0	0.0	1	1.1
Asian supermarket (1.5)	1	0.9	0	0,0	1	1.1
Supermarket (1.8)	8	7.0	3	10.7	5	5.7
Fish shop (2.8)	2	1.7	0	0.0	2	2.3
Green grocer (4.8)	3	2.6	2	7.1	1	1.1
Total	115	100	28	100	87	100

Bold: *p* < 0.05, Fisher’s exact test.

**Table 2:** Assortment, instore promotions and advertisements in public spaces of ready-to-eat foods offered in the school food environment.

Food Category	Assortment ^a^		Discounted Assortment ^a^		Public Advertisements ^b^	
	*n*	%	*n*	%	*n*	% of Total Advertisements
Unhealthy food products	-	-	-	-	83	58.0%
Savory pastries	62	53.9%	34	29.6%	21	13.0%
Fried snacks and fries	27	23.5%	15	13.0%	8	5.0%
Sweet and savory snacks (candy, crisps and chocolate)	58	50.4%	29	25.2%	12	7.5%
Hamburger/kebab	36	31.3%	18	15.7%	16	9.94%
Pizza	8	7.0%	6	5.2%	7	4.35%
Sugar-sweetened beverages	97	84.3%	32	27.8%	18	11.20%
Sport and energy drinks	50	43.5%	7	6.1%	1	0.62%
Healthy food products	-	-	-	-	22	13.66%
Fruit	27	23.5%	16	13.9%	4	2.48%
Light (soft) drinks	89	77.4%	31	27.0%	5	3.11%
Water	77	67.0%	4	3.5%	1	0.62%
Vegetable snacks	14	12.2%	7	6.1%	6	3.73%
Dairy (without added sugar)	22	19.1%	1	0.9%	6	3.73%
Other (not ready to eat)	-	-	-	-	38	26.6%

^a^ Percentage shows the specified product as a proportion of the total number of shops in school food environments open during school hours (*n* = 115). ^b^ Percentage shows the advertisement of the specified product as a proportion of the total number of advertisements in the school food environment (*N* = 143).

**Table 3:** Differences in ready-to-eat food assortment, discounts and advertisements for commercialization and neighborhood socioeconomic status.

	Assortment		In-Store Promotion		Advertisements in Public Space	
	Neighborhood Socioeconomic Status					
	Lower	Higher	Lower	Higher	Lower	Higher
**Unhealthy (in % shops for sale)**						
Savory pastries	**37.0**	**59.8**	21.4	32.2	25.0	12.1
Fried snacks and fries	37.0	19.5	**25.0**	**9.2**	7.5	5.5
Sweet and savory snacks (candy, crisps and chocolate)	40.7	54.0	35.7	21.8	15.0	6.6
Hamburgers/kebab	33.3	29.9	7.1	18.4	**22.5**	**7.7**
Pizza	11.1	4.6	10.7	3.4	5.0	4.4
Sugar-sweetened beverages	**96.3**	**80.5**	32.2	26.4	**22.5**	**6.0**
Sport and energy drinks	**66.7**	**35.6**	**14.3**	**3.4**	2.5	0.0
**Healthy**						
Fruit	18.5	25.3	17.9	12.6	2.5	3.3
Lite (soft) drinks	88.9	73.6	28.6	26.4	**7.5**	**1.1**
Water	81.4	62.1	0.0	4.6	2.5	0.0
Vegetable snacks	11.1	12.6	3.6	6.9	**10.0**	**2.2**
Dairy (without added sugar)	17.9	25.3	10.7	3.4	**7.5**	**1.1**

Bold: *p* ≤ 0.05, Fisher’s exact test.

## Appendix A. School Food Environmental Checklists

**Checklist—Ready-to-eat assortment for sale per retail outlet**		
Retail outlet: Locatus ID:		
If not correct in Locatus enter name, type and ID and make picture in which location tag is on		
Open during school hours (10 a.m.–15 p.m.) □ **yes** □ **no** (yes—continue/no—stop audit)		
**Unhealthy products (ready-to-eat)**	**For sale**	**Sales promotion**
Savory pastries	□ **yes **□ **no**	□ **yes **□ **no**
Fried snacks and fries	□ **yes **□ **no**	□ **yes **□ **no**
Sweet and savory snacks (candy, crisps and chocolate)	□ **yes **□ **no**	□ **yes **□ **no**
Hamburgers/kebab	□ **yes **□ **no**	□ **yes **□ **no**
Pizza	□ **yes **□ **no**	□ **yes **□ **no**
Sugar sweetened beverages	□ **yes **□ **no**	□ **yes **□ **no**
Sport and energy drinks	□ **yes **□ **no**	□ **yes **□ **no**
Other, namely…	□ **yes **□ **no**	□ **yes **□ **no**
**Healthy products (ready-to-eat)**	**For sale**	**Sales promotion**
Fruit (and fruit salad)	□ **yes **□ **no**	□ **yes **□ **no**
Light (soft) drinks	□ **yes **□ **no**	□ **yes **□ **no**
Water	□ **yes **□ **no**	□ **yes **□ **no**
Vegetable snacks	□ **yes **□ **no**	□ **yes **□ **no**
Non-sugared dairy	□ **yes **□ **no**	□ **yes **□ **no**
Other, namely	□ **yes **□ **no**	□ **yes **□ **no**

**Checklist—Public food advertisements**	
ID:	
Type of advertisement:	
Are there foods or drinks promoted? □ **yes** □ **no** (yes—continue/no—stop audit)	
Ready to eat product □ **yes** □ **no**	
Take a picture of the food advertisement □ done	
**Select the advertised food or drink from the list below ➔**	
**Unhealthy products**	**Healthy products**
□ Savory pastries	□ Fruit (or fruit salad)
□ Fried snacks and fries	□ Lite/diet (soft) drinks
□ Sweet and savory snacks (candy, crisps and chocolate)	□ Water
□ Hamburgers/kebab	□ Vegetable snacks
□ Pizza	□ Non-sugared dairy
□ Sugar sweetened beverages	
□ Sport and energy drinks	
□ not-ready to eat, namely	□ not-ready to eat, namely

## Appendix B. Food Outlet Availability, Differentiated by Neighborhood Socioeconomic Status and Commercialization

**Table:**

	Total		Neighborhood Socioeconomic Status				Neighborhood Commercialization			
			Low		High		Low		High	
Type (Score)	*N*	%	*N*	%	*N*	%	*N*	%	*N*	%
Fast-food (−4.9)	18	15.7	**8**	**28.6**	**10**	**11.5**	3	18.8	15	15.3
Grillroom(−4.8)	4	3.5	2	7.1	2	2.3	0	0.0	4	4.1
Liquor store (−4.6)	6	5.2	2	7.1	4	4.5%	1	6.3	5	0.0
Petrol station (−4.5)	1	0.9	0	0.0	1	1.1	1	6.3	0	0.0
Chocolate store (−4.3)	3	2.6	0	0.0	3	3.4	0	0.0	3	3.1
Drugstore (−4.0)	9	7.8	4	14.3	5	5.7	2	12.5	7	7.1
Ice-cream store (−3.9)	2	1.7	1	3.6	1	1.1	0	0.0	2	2.0
Take away (−3.7)	8	7.0	1	3.6	7	8.0	1	6.3	7	7.1
Café-Restaurant (−1.9)	6	5.2	0	0	6	6.8	0	0.0	6	6.1
Lunchroom (−1.5)	20	17.4	**1**	**3.6**	**19**	**21.8**	1	6.3	18	18.4
Butcher (−0.4)	2	1.7	0	0.0	2	2.3	1	6.3	1	1.0
Delicacies (0.2)	3	2.6	0	0.0	3	3.4	0	0.0	3	3.1
Mini super (0.3)	4	3.5	1	3.6	3	3.4	1	6.3	3	3.1
Reform shop (1.0)	2	1.7	0	0.0	2	2.3	0	0.0	2	2.0
Bakery (1.2)	8	7.0	2	7.1	6	6.9	1	6.3	7	7.1
Coffee/tea store (1.3)	4	3.5	0	0.0	1	1.1	0	0.0	4	4.1
Asian Supermarket (1.5)	1	0.9	0	0.0	1	1.1	0	0.0	1	1.0
Supermarket (1.8)	8	7.0	3	10.7	5	5.7	2	12.5	6	6.1
Fish shop(2.8)	2	1.7	0	0.0	2	2.3	1	6.3	1	1.0
Green grocer (4.8)	3	2.6	2	7.1	1	1.1	1	6.3	2	2.0
Total	114	100	28	100	87	100	17	100	97	100

Bold: *p* ≤ 0.05, Fisher’s exact test.

## Appendix C. Food Assortment, In-Store Food Promotions and Food Advertisements in Public Space, Differentiated by Neighborhood Socioeconomic Status and Commercialization.

**Table:**

	Ready-to-eat assortment				In-Store Promotion				Advertisements Public Space			
	Commercialization		Neighborhood Socioeconomic Status		Commercialization		Neighborhood Socioeconomic Status		Commercialization		Neighborhood Socioeconomic Status	
	Lower	Higher	Lower	Higher	Lower	Higher	Lower	Higher	Lower	Higher	Lower	Higher
# of Shops	(*N* = 16)	(*N* = 98)	(*N* = 27)	(*N* = 87)	(*N* = 16)	(*N* = 98)	(*N* = 27)	(*N* = 87)	(*N* = 51)	(*N* = 88)	(*N* = 41)	(*N* = 96)
**Unhealthy (% shops for sale)**												
Savory Pastries	56.3	53.6	**37.0**	**59.8**	50.0	26.8	21.4	32.2	18.8	14.5	25.0	12.1
Fried Snacks and fries	31.3	21.6	37.0	19.5	12.5	13.4	**25.0**	**9.2**	8.3	4.8	7.5	5.5
Sweet and savory snacks (Candy, crisps and Chocolate)	43.8	51.5	40.7	54.0	18.8	26.8	35.7	21.8	6.3	10.8	15.0	6.6
Hamburgers/kebab	31.1	29.9	33.3	29.9	25.0	14.4	7.1	18.4	18.8	8.4	**22.5**	**7.7**
Pizza	0.0	7.2	11.1	4.6	0.0	5.2	10.7	3.4	6.3	3.6	5.0	4.4
Sugar sweetened beverages	87.5	83.5	96.3	80.5	**56.3**	**23.7**	32.2	26.4	16.7	8.4	**22.5**	**6.0**
Sport and Energy drinks	50.0	52.3	**66.7**	**35.6**	6.3	6.2	14.3	3.4	0.0	1.2	2.5	0.0
**Healthy**												
Fruit	37.5	20.6	18.5	25.3	**31.3**	**11.3**	17.9	12.6	0.0	4.8	2.5	3.3
Lite (soft) drinks	87.5	75.3	88.9	73.6	**50.0**	**23.7**	28.6	26.4	2.1	3.6	7.5	1.1
Water	62.5	67.0	81.4	62.1	0.0	4.1	0.0	4.6	0.0	1.2	2.5	0.0
Vegetable snacks	18.8	11.3	11.1	12.6	6.3	6.2	3.6	6.9	4.2	4.8	10.0	2.2
Dairy	31.3	21.4	17.9	25.3	6.3	5.1	10.7	3.4	0.0	4.8	7.5	1.1

Bold: *p* ≤ 0.05, Fisher’s exact test.
